# Action force modulates action binding: evidence for a multisensory information integration explanation

**DOI:** 10.1007/s00221-020-05861-4

**Published:** 2020-07-02

**Authors:** Liyu Cao, Michael Steinborn, Wilfried Kunde, Barbara Haendel

**Affiliations:** Department of Psychology (III), Julius-Maximilians-Universität Würzburg, 97070 Würzburg, Germany

**Keywords:** Action binding, Force, Somatosensory feedback, Multisensory processing

## Abstract

Action binding refers to the observation that the perceived time of an action (e.g., a keypress) is shifted towards the distal sensory feedback (usually a sound) triggered by that action. Surprisingly, the role of somatosensory feedback for this phenomenon has been largely ignored. We fill this gap by showing that the somatosensory feedback, indexed by keypress peak force, is functional in judging keypress time. Specifically, the strength of somatosensory feedback is positively correlated with reported keypress time when the keypress is not associated with an auditory feedback and negatively correlated when the keypress triggers an auditory feedback. The result is consistent with the view that the reported keypress time is shaped by sensory information from different modalities. Moreover, individual differences in action binding can be explained by a sensory information weighting between somatosensory and auditory feedback. At the group level, increasing the strength of somatosensory feedback can decrease action binding to a level not being detected statistically. Therefore, a multisensory information integration account (between somatosensory and auditory inputs) explains action binding at both a group level and an individual level.

**Electronic supplementary material:**

The online version of this article (10.1007/s00221-020-05861-4) contains supplementary material, which is available to authorized users.

## Introduction

When an action foreseeably produces a slightly delayed action effect (e.g., a keypress triggers a 250 ms delayed sound), the reported time of the action shifts forward in time towards the delayed action effect. This is referred to as the action binding effect. It is typically computed by subtracting the reported action time in a condition without a sound (action only condition, AO) from the reported action time when a sound is produced by the action (action sound condition, AS). Together with outcome binding, i.e., the shift of the reported time of the action effect towards the action, the action binding effect forms the so-called intentional binding effect (Haggard et al. 2002). Despite the wide use of the intentional binding effect as an implicit measure of sense of agency, the mechanism of the intentional binding effect is still a subject of debate (Buehner 2012; Buehner and Humphreys 2009; Haggard 2017; Kirsch et al. 2019; Moore and Obhi 2012; Moore et al. 2010; Suzuki et al. 2019).

Imagine making a keypress (e.g., while typing) and afterwards giving an estimate of the keypress time. Although a keypress spans a time period, no one raises any doubt about the existence of a time point of the keypress. This is presumably because one keypress always serves one functional purpose, and people often take the moment when the functional purpose is achieved as the keypress time. The achievement of the functional purpose is usually displayed as some sort of immediate sensory feedback (e.g., I immediately see the letters, while I am typing). The reported keypress time, or the psychological keypress time (cf. the perceptual centre of a sound, Morton et al. 1976), therefore, must take contribution from the keypress-related sensory feedback, which lays the foundation of the action binding effect (Haggard et al. 2002; Stetson et al. 2006). However, when there is a long delay before the keypress and keypress-related sensory feedback (e.g., a sound is always played 3 s after making a keypress), how should the keypress time be determined? One possibility is to rely on the intention of the keypress (or the ‘command to move’; Mccloskey et al. (1983). The perceived time of motor intention is clearly before the actual movement onset measured from EMG (electromyogram) (Libet et al. 1983; Mccloskey et al. 1983). The reported keypress time, however, is most likely later than the EMG measured movement onset (Haggard et al. 2002; Haggard and Cole 2007; Wolpe et al. 2013). Note that the EMG measured movement onset time is much earlier than the computer-registered keypress onset time [by about 30–50 ms as reported in Haggard and Eimer (1999)]. Therefore, it is highly unlikely that participants report the motor intention time as the keypress time. The other possibility is to rely on the somatosensory feedback, which is salient enough to provide information about the keypress time. However, the role of somatosensory feedback has been largely ignored as a possible explanatory variable in action binding (cf. Aschersleben and Prinz 1995; Haggard and Cole 2007). In the present paper, we study the influence of the strength of somatosensory feedback, which varies naturally with keypress force, on action binding. There are two potential influences.

**Fig. 1:**
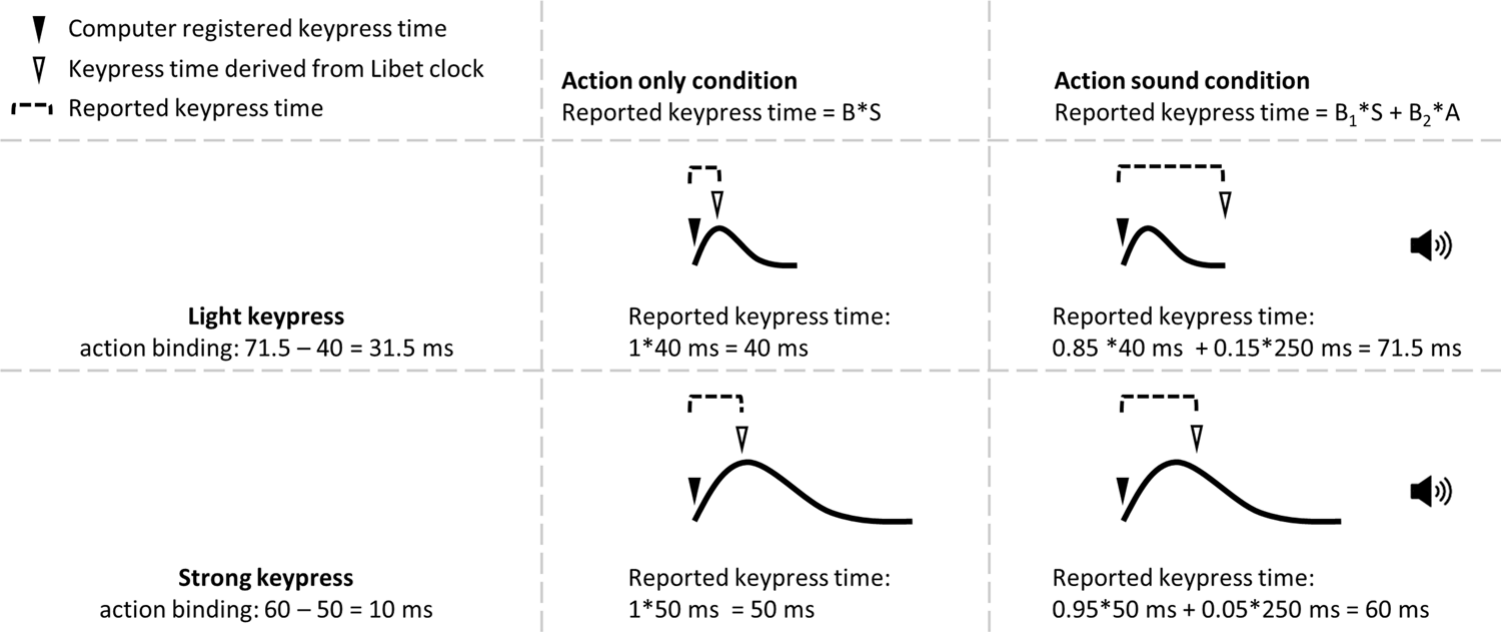
Schematic illustration of the influence of keypress peak force. The solid lines illustrate the force profile of a keypress. In the action only condition, the reported keypress time (calculated as the difference between the keypress time derived from Libet clock and the computer-registered keypress time) is positively correlated with to the strength of somatosensory feedback, i.e., the keypress peak force. *S* shows the keypress time extracted from the somatosensory feedback, and *B* is the weighting factor. In the action only condition, we assume that the reported keypress time takes all the contribution from the somatosensory feedback for simplicity (*B* = 1). In the action sound condition, the reported keypress time is shaped by both somatosensory feedback and auditory feedback (*B*_1_*S + *B*_2_*A; *B*_1_ + *B*_2_ = 1). The stronger the keypress, the more weight is given to the somatosensory feedback, i.e., a larger *B*_1_. The above example clearly indicates that a stronger keypress should lead to a smaller action binding effect. The numbers and equations in the example are only given to illustrate the rationale behind the study. The duration bars are only relative

First, the point in time of the somatosensory cue, which might be used to judge keypress time, varies with keypress force. Figure 1 shows sample force trajectories of light and strong keypresses. It is apparent that most of the features that describe the force curve and thus somatosensory feedback, such as peak force, or the point of strongest acceleration or return to baseline, arise later with a strong than a light keypress. Assuming that actors rely on some of these features (or combination thereof) as cues of keypress time, strong keypresses should be judged to occur later than light keypresses. Thus, in the AO condition, where the somatosensory input might be the most salient sensory input in determining the action time, forceful keypresses should appear to occur later in time, which should work towards a smaller action binding effect with forceful keypresses.

Second, in the AS condition, an additional sensory input from the auditory domain can influence the judgment of action time, i.e., the action time judgment will be shaped by both somatosensory and auditory inputs. Therefore, the result of action time judgement depends on the relative strength of the two signals. A strong keypress will gain more weight for the somatosensory cue in determining the action time in the competition with a constant auditory cue as compared to a weak keypress, thus leading to an action time judgment closer to the somatosensory cue (further away from the auditory cue), i.e., an earlier action time (assuming that a strong keypress gains more influence on reported keypress time from the multisensory competition process than from the change of somatosensory input alone as described in the above paragraph). In other words, a stronger somatosensory cue should be less vulnerable to being attracted towards a later auditory cue in a typical action sound condition (see an illustration in Fig. 1). This would work towards a *weaker* action binding effect with forceful keypresses.

To summarise the above two points, a strong somatosensory input should be associated with a late-reported action time when there is no competing auditory cue. Additionally, a strong somatosensory cue should be less prone to influences from a secondary auditory cue (due to sensory information weighting), i.e., stick closer to the action time as indicated by the somatosensory cue. In this report, we provide evidence that somatosensory feedback is actually used in judging action time and that action binding can be interpreted as a multisensory information integration process between the somatosensory and the auditory input. We do so in three steps. First, we show a group-level correlation between spontaneously varying peak force of actions (keypresses) and the reported action time, which provides crucial evidence for the involvement of the somatosensory feedback in judging action time. Second, we show that the influence of peak force on the reported action time changes with additional auditory feedback, suggesting a competition between somatosensory and auditory feedback in shaping the action time judgment. Finally, we show that the action binding effect can be predicted by this competition account, by showing that experimentally increased keypress peak forces lead to decreased action binding.

## Methods

### Participants

60 participants were recruited in the first study (mean age = 26.6, SD = 8.9, 42 females). In the second study in which the keypress peak force was controlled, another 40 participants were recruited (mean age = 25.9, SD = 6.4, 35 females). All participants provided written informed consent prior to the study and received monetary compensation after the study. The study was approved by the local ethics committee (Institute of Psychology, Faculty for Human Sciences, University of Würzburg; project number: GZ 2018-27) and followed the Declaration of Helsinki and the European data protection law (GDPR).

### Sample size and statistical power

The first study was designed to look for evidence for the involvement of somatosensory information in shaping the reported action time. For this purpose, we combined two datasets (30 participants each) with a similar action binding testing set-up to maximise the power. We tested as many participants as the lab resources allowed. A post hoc analysis showed that 35 participants were needed to achieve a statistical power of 0.8 to detect the group-level correlation between keypress peak force and reported keypress time in the AO condition, and that 39 participants were needed in the AS condition. The power analysis was performed through bootstrapping, i.e., randomly sampling from the existing participants (without replacement). We note that the result from the post hoc power analysis is bounded by the sample tested in the study. Therefore, reporting the statistical power only serves the purpose of guiding future research. For the second study, a similar power analysis was run using the data acquired from the first study through bootstrapping (only including the 42 participants used in the final data analysis; see “Data analysis” section). A linear regression analysis was performed to find the relationship between the reported keypress time and the keypress peak force, separately for each condition of each participant. A change of reported keypress time due to a force increase of 2 N was obtained from the regression analysis for each condition of each participant. The probability of detecting the predicted interaction effect between keypress peak force and action binding (*p* < 0.05) was estimated by randomly sampling (without replacement) from the 42 participants. The sample size in the bootstrap analysis increased from 20 to 42 (step size: 1), and 1000 repetitions were performed for each sample size. Supplementary Figure 1 shows the results of the bootstrap analysis. With 40 participants, the detection power was 1. Therefore, we included 40 participants in the second study. Due to a without-replacement sampling procedure and a total number of 42 participants, it should be noted that there is only 1 possibility when the sample size is 42 in the bootstrap analysis, i.e., all 1000 repetitions have the same data. Similarly, there are 42, 42 × 41 possibilities when the sample size is 41, 40, respectively. Therefore, a very high statistical power with the sample size in the bootstrap analysis approaching the total number of available participants should be interpreted with caution.

### Task, design, and procedure

The experiment was conducted in a light room, and participants completed the task in front of a computer (viewing distance was around 50 cm). The tasks in both studies were quite similar. We first describe the common set-up of both studies and then detail the difference. Participants reported their keypress time in an Action Sound condition and an Action Only condition using the Libet clock method (Libet et al. 1983). In the AS condition, participants made a voluntary keypress while watching a clock with a rotating hand in the centre of the screen. The clock hand rotated clockwise 2° per refresh frame, and the screen refresh rate was 100 Hz, i.e., the rotating hand had a period of 1800 ms. After the keypress, a sound (1000 Hz; 50 ms long; 5 ms rise/fall envelop; comfortable volume level) was presented binaurally via headphones with a delay of 250 ms (the empirically measured delay had a mean of 253 ms with a range of [252 254] ms; 100 trials measurement; we still refer to the delay as 250 ms in the manuscript instead of 253 ms). Participants were asked to report their keypress time by adjusting the clock hand to the position when the keypress was made. In the AO condition, everything was the same as the AS condition except that no sound was played after the keypress. There were 60 trials in each condition. Participants were given a few trials of practise before the formal testing started.

**Fig. 2:**
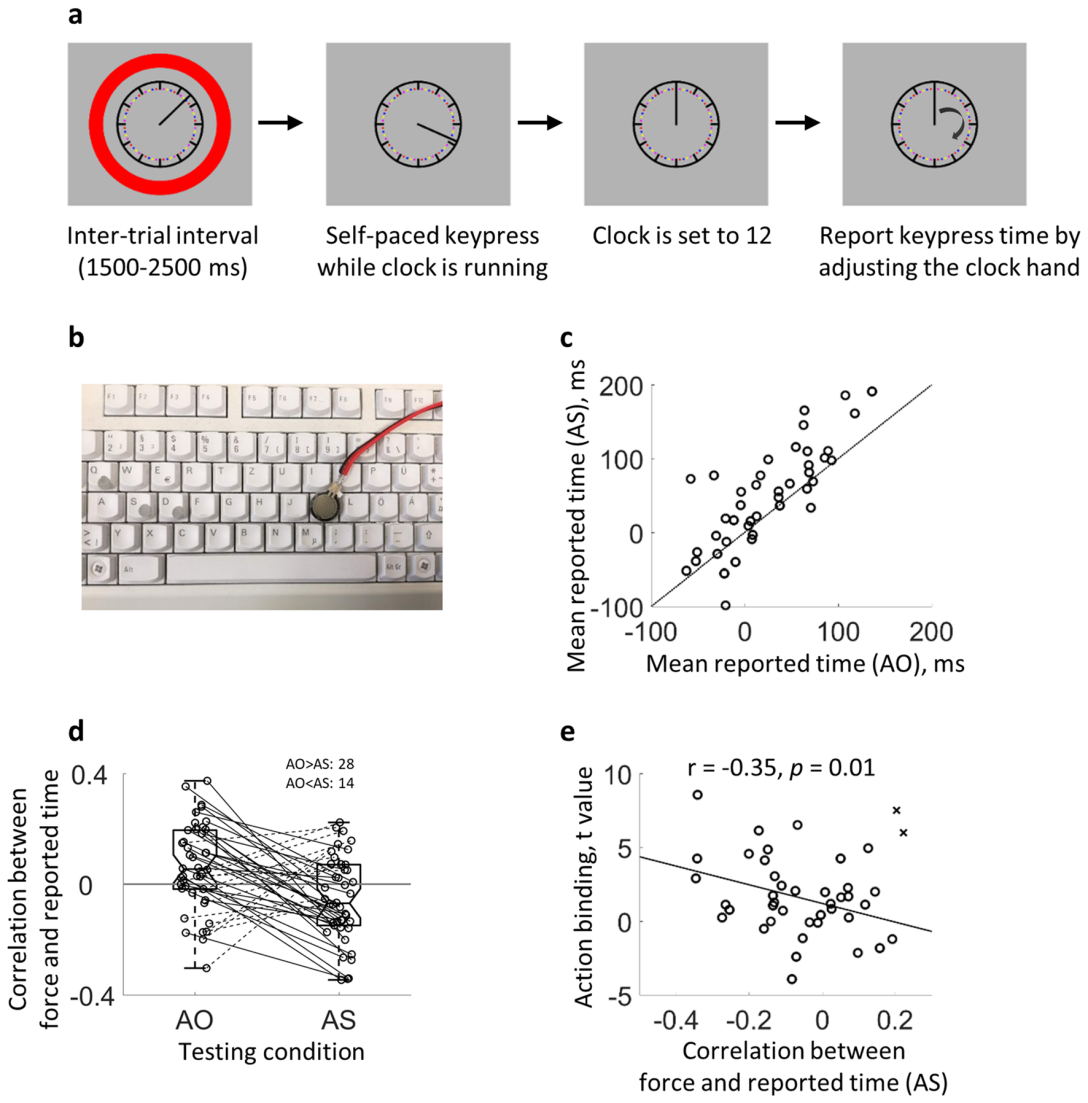
Action binding in the first study. **a** A schematic illustration of a trial. During the inter-trial interval, a red circle surrounds the rotating clock, and no responses are required. After the disappearance of the red circle, participants make a keypress at a self-chosen time while being encouraged not to respond immediately after the disappearance of the red circle. After the keypress, the clock hand continues to rotate for a random period between 1000 and 1500 ms before it stops at the 12 o’clock position. Participants adjust the clock hand position to the keypress time point. **b** The force sensor and the keyboard used in the study. **c** The reported keypress time in the AS and the AO conditions. Each circle represents a participant and the diagonal is superimposed. The majority of data points lie above the diagonal, thus demonstrating an action binding effect. **d** Correlation coefficients between the keypress peak force and the reported keypress time in the AO condition and AS condition. Each circle represents a participant. Lines link the data points belonging to the same participant (solid line: AO > AS; dashed line: AO < AS). The central mark of the boxplot is the median, the edges of the box are the 25th and 75th percentiles, and the whiskers extend to the most extreme data points within 1.5 times of interquartile range. The horizontal grey line marks the level of 0 correlation. **e** Scatter plot between the size of action binding effect and the correlation coefficient between the keypress peak force and the reported keypress time in the AS condition. A large action binding effect is associated with a large *t* value (*y* axis). A negative correlation coefficient is associated with an engagement of the multisensory information integration process in judging action time. Note that two outliers are marked with a cross

In each trial, participants were asked to fixate on the rotating clock in the centre of the screen (Fig. 2a). During the inter-trial interval (randomly sampled between 1500 and 2500 ms), the clock hand was also rotating but a red circle surrounded the clock (indicating that no keypress should be made). When the inter-trial interval ended, the red circle was removed, and participants made a voluntary keypress with the right index finger via the key ‘K’ on a standard keyboard at a self-chosen time. They were required not to respond in a stereotyped way (e.g., always making the keypress at the same clock hand position) or at a predecided clock hand position. These requirements were made as close to the original study of intentional binding as possible (Haggard et al. 2002). They were also told not to make the keypress immediately after the offset of the red circle and that the keypress should be made briskly and as quietly as possible. Depending on the condition, a 250 ms delayed sound was played after the keypress. The clock hand continued rotating for another period randomly sampled between 1000 and 1500 ms and then stopped at the 12 o’clock position. Participants then manually adjusted the clock hand position via pressing ‘S’ and ‘D’ on the keyboard until the clock hand was at the position which matched the keypress time. Each ‘S’/‘D’ press moved the clock hand 2° anticlockwise/clockwise, which means that the resolution of the reported keypress time was 10 ms. Participants were asked to estimate the keypress time as accurately as possible. After the keypress time estimation, participants pressed key ‘A’ to proceed to the next trial.

Data for the first study were combined from two separate datasets. In both datasets, there were other manipulations including the AS and AO conditions introduced above. However, only the AS and AO conditions were the focus of the current report. The complete task involved in both datasets is given below for completeness.

In dataset 1 (30 participants; 14 included in the final analysis, see “Data analysis” section), participants completed the AS and AO conditions in separate blocks. The block order was randomised. In addition, participants completed a variation of the task in which they were asked to make the keypress with a strong force in both conditions. The order between the relevant task and the variation of the task was counterbalanced among participants.

In dataset 2 (30 participants; 28 included in the final analysis, see “Data analysis” section), four conditions were involved. In addition to the AS and AO conditions, there was a Sound Only condition in which a sound (without a keypress) was presented together with the rotating clock, and participants later reported the time of the sound play. There was also a second Action Sound condition, but required a report of the sound play time. The Sound Only condition and the second Action Sound condition were typically used to measure the outcome binding effect, which formed the intentional binding effect together with action binding. The four conditions were presented in separate blocks with 60 trials each. The order of the conditions was randomised except that the Sound Only condition was always after the AO condition, so that the time when the sound was played during a trial in the Sound Only condition can match the time when the keypress was made during a trial in the AO condition. In a variation of the task, the same four conditions were included except that participants were required to report the time when they started to make the keypress (i.e., the time point when the finger started to apply force on the key) in the AS and AO conditions. The reported keypress onset time from the variation of the task was reported in the results section. The variation task was always run later than the normal task.

In the second study, both AS and AO conditions were tested under a light keypress condition and a strong keypress condition, i.e., a 2 (AS vs. AO) by 2 (light vs. strong) design. The four conditions were tested in separate blocks of 60 trials. The block order was randomised. In each trial, the keypress force was recorded during the whole keypress response period, i.e., from the offset of the red circle to the onset of the frame requiring reporting the keypress time. If the keypress peak force recorded during this period was within a pre-specified range, the participant continued to report the keypress time. If the keypress peak force was out of the range, the trial was repeated after a message telling participants to press lighter or harder in the next try. The accepted peak force range was [350 450] (raw force measurement output unit, see below; [1.06 1.31] in N) in the light keypress condition and [700 800] ([2.57 4.01] in N) in the strong keypress condition. The range of light force was selected to match the average keypress peak force in the first study (around 1.2 N). The range of strong force was selected to be higher than the light force range but clearly below the force measurement limit of the sensor (the limit is 1023 in raw unit).

The force value of each keypress was recorded with a force sensing resistor (FSR; model 400, Interlink Electronics Inc., USA; 518 Hz sampling rate; Fig. 2b). The FSR has a circular shape with a diameter of about 1.5 cm, and it is very thin and light. The FSR was directly attached to the top of the ‘K’ key on the keyboard, so that the force of each keypress could be measured. The FSR measures force by reducing its resistance with increased force applied on its surface, (see the FSR datasheet: https://www.interlinkelectronics.com/data-sheets). Since the relationship between the FSR resistance and the applied force is not linear, the output of the FSR (in our case, values between 0 and 1023 in steps of 1) changes monotonically but not linearly with the applied force in Newton. We empirically measured the relationship between the applied force and the FSR output and fitted a polynomial function to convert the unit of FSR output to Newton for the data analysis.

The task presentation was controlled by the Matlab (The MathWorks Inc., USA) toolbox Psychtoolbox-3 (Kleiner et al. 2007), and the data collection was controlled by the software Lab Streaming Layer (https://github.com/sccn/labstreaminglayer).

### Data analysis

For each trial, the reported keypress time was referenced to the computer-registered time of keypress onset (the keypress time), i.e., the reported keypress time = the time point of the reported clock hand position—the keypress time. The peak force of each keypress was searched in an 800 ms time window starting from the keypress time (visually checked that within this time window, the peak force of each trial can be accurately detected for all participants). The maximum force intensity within this window was defined as the keypress peak force.

Trials were rejected based on the reported keypress time and the recorded keypress force parameters. For each testing condition of each participant, trials with a reported keypress time that was 450 ms (equivalent to a distance of 90° on the rotating clock) away from the condition median was first rejected. Further outliers of the reported keypress time were rejected if they were more than three standard deviations away from the condition average. Trials were also rejected if no reliable keypress force was recorded (only relevant for the first study) or the keypress peak force was more than three standard deviations away from the condition average. A participant was excluded if less than 30 trials (half of all trials) were left in any of the relevant conditions here (9 participants from the first study and 0 from the second study were excluded). We further excluded the participants whose reported keypress time was more than 100 ms before the keypress time in any condition (see Supplementary Figure 2 for the reported keypress time of all participants in the first study). It might be that those participants used the red circle offset as the cue when reporting the keypress time as their keypress time was very close to the offset of the red circle (9 participants from the first study and 2 participants from the second study). There were three participants whose data were not complete from the second study. Therefore, 42 participants from the first study (60-9-9) and 35 participants from the second study (40-2-3) were included in the final data analysis. Of the remaining participants, the average number of trials included in the data analysis was 55.43 (SD = 5.70) (AO condition, study 1), 55.12 (SD = 7.05) (AS condition, study 1), 57.46 (SD = 4.13) (AO condition, light keypress, study 2), 57.60 (SD = 3.76) (AS condition, light keypress, study 2), 57.74 (SD = 3.19) (AO condition, strong keypress, study 2), and 58.34 (SD = 2.42) (AS condition, strong keypress, study 2). No significant difference in trial number was found between conditions in study 1 or study 2 (all *p* values ≥ 0.2).

The action binding effect was evaluated by a one-tailed within-subjects *t* test comparing the reported keypress time between the AS and the AO conditions. The size of action binding for each participant was represented by the *t* value of a two-sample *t* test between AS and AO condition, i.e., a positive action binding effect was associated with a positive *t* value. The choice of using the t value, instead of the raw difference in reported keypress time between conditions, to represent the size of action binding was due to the fact that *t* values are normalised by the sample variance and, therefore, could represent the individual action binding size more accurately than the raw difference (see Supplementary Figure 3 for a demonstration). A correlation coefficient was computed between the keypress peak force and the reported keypress time for each condition and each participant (skipped Pearson’s correlation) before being subjected to a group-level analysis by comparing the average correlation coefficient to 0 (two-tailed). The group-level distribution of correlation coefficients did not deviate from a normal distribution in both AO and AS conditions as indicated by Lilliforces test (*p* > 0.89 in both conditions) and Jarque–Bera test (*p* > 0.73 in both conditions) (Jarque and Bera 1987; Lilliefors 1967). The same correlation analysis was also performed between the peak force itself and the peak force latency (the duration between the computer-registered keypress point and the peak force point) as a demonstration of the correlation between force parameters. Cross-participant correlations between action binding size and the correlation coefficient between the keypress peak force and the reported keypress time were also evaluated using the skipped Pearson’s correlation for robustness. The skipped Pearson’s correlation analysis first detects bivariate outliers using a boxplot rule and then computes the Pearson’s correlation with the detected outliers left out. A 95% confidence interval of cross-participant correlations was computed using the bootstrap method. The skipped Pearson’s correlation analysis is implemented in a Matab toolbox (Pernet et al. 2013). All the data analyses were performed with Matlab.

## Results

Participants made voluntary keypresses while watching a rotating clock on the screen. After each keypress, the keypress time was reported using the Libet clock method (Libet et al. 1983) (Fig. 1a). In the Action Sound (AS) condition, the keypress triggered a sound with a 250 ms delay. In the Action Only condition, no sound followed the keypress. A significant action binding effect was observed [*t*(41) = 4.01, *p* = 1.25e−4, one-tailed, *d*_*z*_ = 0.62], i.e., the reported keypress time in the AS condition (mean = 50.31 ms, SD = 67.89) was later than in the AO condition (mean = 24.92 ms, SD = 50.97) (Fig. 2c; data points lying above the diagonal show a positive action binding effect).

Among the 42 participants reported above, 28 of them were also asked to report the keypress onset time in a separate test (‘the time point when your finger starts to apply force on the key’). In the AO condition, the reported keypress onset time was − 35.22 ms (SD = 78.85), which was significantly earlier than the reported keypress time [*t*(27) = − 5.28, *p* = 7.19e−6, one-tailed, *d*_*z*_ = − 1.00]. This indicates that the somatosensory feedback must be used as the source of information to judge the keypress time in the AO condition. We then tested if the keypress peak force was related to the reported keypress time. Within each participant, a correlation coefficient was computed between the reported keypress time and the keypress peak force across trials using the skipped Pearson’s correlation (Pernet et al. 2013). The average correlation coefficient of all participants was then compared to 0 (distribution of correlation coefficients in both conditions did not deviate from a normal distribution). In the AO condition, the correlation coefficient was larger than 0 (mean = 0.06, SD = 0.16; *t*(41) = 2.64, *p* = 0.01, two-tailed, *d*_*z*_ = 0.41; Fig. 2d, left), suggesting that on the group level, the keypress-related somatosensory feedback was used in reporting the keypress time: the stronger the keypress, the later the reported keypress time. In the AS condition, however, the mean correlation between the keypress peak force and reported keypress time was smaller than 0 [mean = − 0.06; SD = 0.15; *t*(41) = − 2.34, *p* = 0.02, two-tailed, *d*_*z*_ = − 0.36; Fig. 2d, right]. Thus, the stronger the keypress, the earlier the reported keypress time. Not surprisingly, the average correlation coefficient was significantly smaller in the AS condition than in the AO condition [*t*(41) = − 3.35, *p* = 0.002, two-tailed, *d*_*z*_ = − 0.52].

The opposite relationship between the keypress peak force and the reported keypress time in the AO and AS conditions may be interpreted in the following way. In the AO condition, the somatosensory feedback (represented by the keypress peak force in our case) is a major source of sensory information that can be used for judging the keypress time. Therefore, the reported keypress time is late when the somatosensory cue is late. A possible explanation could be that higher peak forces take longer time to develop. This is confirmed by our data, showing that the latency of the keypress peak force was positively correlated with the keypress peak force. This within-participant correlation was significantly larger than 0 in both AO [mean = 0.23, SD = 0.28; *t*(41) = 5.32, *p* = 4.03e−6, two-tailed, *d*_*z*_ = 0.82] and AS [mean = 0.24, SD = 0.25, *t*(41) = 6.30, *p* = 1.64e−7, two-tailed, *d*_*z*_ = 0.97] conditions.

In the AS condition, the auditory input competes with the somatosensory feedback in shaping the reported keypress time, as the auditory input is also a source of information about the keypress time (a multisensory information integration process). The average keypress peak force latency from the keypress onset was 56.46 ms (SD = 17.53; over participants), whereas the sound was constantly delayed for 250 ms, and therefore arrived later than the keypress peak force. The stronger the keypress, the higher the weight of the somatosensory information compared to the auditory information. A strong keypress can thus lead to a reported keypress time closer to the somatosensory feedback. Since the somatosensory feedback occurs much earlier than the auditory feedback, a stronger keypress results in an earlier report. Therefore, a negative correlation between the keypress peak force and the reported keypress time should be observed if the multisensory information integration process is engaged (see also Fig. 1). The more negative the correlation, the stronger the engagement of the multisensory information integration process. Only when the multisensory information integration process is engaged in judging the keypress time in the AS condition (i.e., auditory information is considered in addition to the somatosensory information), an action binding effect can be found. Supporting this view, a negative correlation across participants was found between the correlation coefficient in the AS condition and the size of the action binding effect (skipped Pearson’s *r* = − 0.35, *p* = 0.01, one-tailed, 95% CI = [− 0.60 − 0.03]; Fig. 2e).^1^ Thus, the negative correlation was in principle in agreement with the idea that participants relying more on the multisensory information integration process when judging the keypress time in the AS condition showed a larger action binding effect (the exact weight assigned to the somatosensory information in the AS condition is not considered here).

If the peak force of a keypress is functional in determining the reported keypress time, as is speculated above, experimentally manipulating the keypress peak force should lead to predictable changes of the action binding effect. Specifically, a light keypress in both AO and AS conditions should be ideal for the detection of action binding, as a light keypress should lead to an early reported keypress time in the AO condition and a late-reported keypress time in the AS condition (Fig. 1). Conversely, a strong keypress in both AO and AS should work towards reducing the action binding effect as compared to a light keypress, if an action binding effect is still detectable at all.

A new group of participants were tested with the keypress peak force controlled. Each participant completed both AO and AS conditions under light and strong keypresses. The mean peak force under light keypress was not significantly different [*t*(34) = 0.99, *p* = 0.33, two-tailed, *d*_*z*_ = 0.17] between the AO condition (mean = 1.18 Newton; SD = 0.01) and the AS condition (mean = 1.18 Newton; SD = 0.02). It was also not significantly different under strong keypress [*t*(34) = 1.28, *p* = 0.21, two-tailed, *d*_*z*_ = 0.22; AO condition: mean = 3.12 Newton; SD = 0.10; AS condition: mean = 3.09 Newton; SD = 0.10]. No significant interaction effect was found with a 2 (AO vs. AS) by 2 (light vs. strong keypress) within-subjects ANOVA comparing the peak force [*F*(1,34) = 1.21, *p* = 0.28, *η*_*p*_^*2*^ = 0.03] (see Supplementary Figure 4 for the average force trajectories in all conditions).

**Fig. 3:**
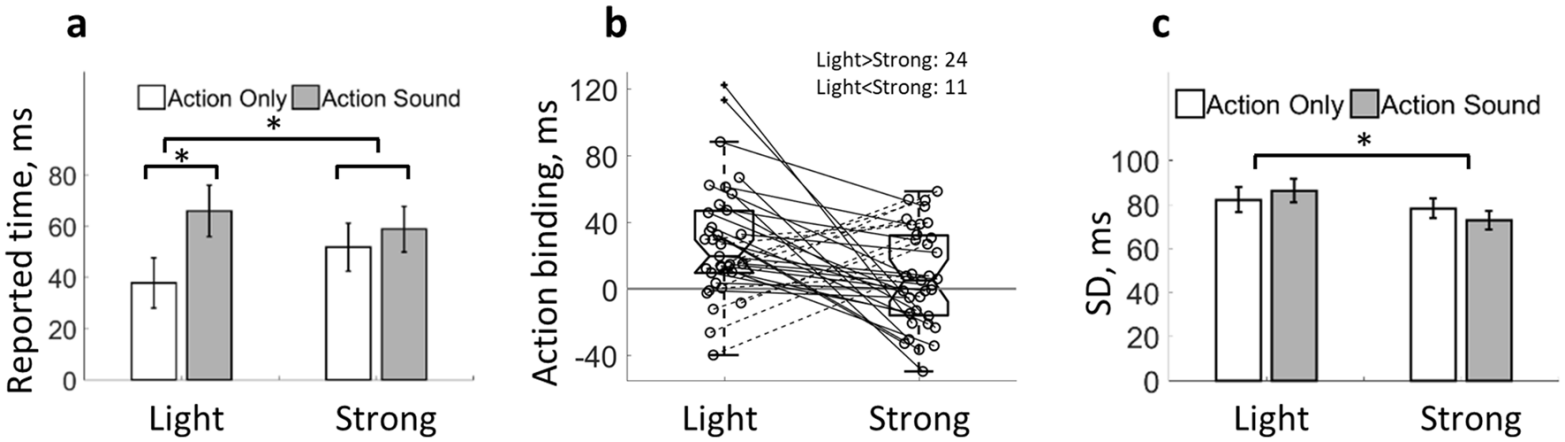
Action binding with controlled keypress peak force. **a** The action binding effect is significantly larger under light keypress (action binding is significant) than under strong keypress (no significant action binding). Bars represent ± 1 standard error. **b** Individual action binding effect (the difference in reported keypress time between AO and AS conditions). Each circle represents a participant. Lines link the data points belonging to the same participant (solid line: light > strong; dashed line: light < strong). The central mark of the boxplot is the median, the edges of the box are the 25th and 75th percentiles, and the whiskers extend to the most extreme data points within 1.5 times the interquartile range. Data points outside 1.5 times the interquartile range are marked with crosses. The horizontal grey line marks 0 action binding. **c** The standard deviation of reported keypress time is lower under stronger keypress than under light keypress. Bars represent ± 1 standard error

A 2 (AO vs. AS) by 2 (light vs. strong keypress) within-subjects ANOVA was then performed comparing the reported keypress time. Reported keypress time was smaller in the AO condition as compared to the AS condition indicating an action binding effect [*F*(1,34) = 28.71, *p* = 5.88e−6, *η*_*p*_^*2*^ = 0.46; Fig. 3a, b]. More importantly, a significant interaction was also found [*F*(1,34) = 5.73, *p* = 0.02, *η*_*p*_^*2*^= 0.14]. The action binding effect was much stronger under light keypress [*t*(34) = 4.73, *p* = 1.89e−5, one-tailed, *d*_*z*_ = 0.80; AO: mean = 37.81 ms, SD = 57.81; AS: mean = 65.83 ms, SD = 59.19] than under strong keypress [*t*(34) = 1.41, *p* = 0.08, one-tailed, *d*_*z*_ = 0.24; AO: mean = 51.74 ms, SD = 55.12; AS: mean = 58.75 ms, SD = 52.80]. No significant main effect was found between light and strong keypresses [*F*(1,34) = 0.47, *p* = 0.50, *η*_*p*_^*2*^ = 0.01].

In addition, the standard deviation of reported keypress time was calculated for each condition of each participant (Fig. 3c). A 2 (AO vs. AS) by 2 (light vs. strong keypress) within-subjects ANOVA revealed a significant main effect of keypress force [*F*(1,34) = 6.85, *p* = 0.01, *η*_*p*_^*2*^ = 0.17], indicating that the precision of reported keypress time was higher under strong keypress than under light keypress. No other effects were significant [AO vs. AS: *F*(1,34) = 0.11, *p* = 0.74, *η*_*p*_^*2*^ = 0.003; interaction: *F*(1,34) = 3.59, *p* = 0.07, *η*_*p*_^*2*^ = 0.10]. The group average standard deviation of reported keypress time was: AO light 82.03 (SD = 34.04), AS light 86.19 (SD = 32.10), AO strong 78.16 (SD = 26.64), and AS strong 72.74 (SD = 24.60).

## Discussion

Our data show that the keypress peak force has an influence on the reported keypress time. The influence has opposite signs depending on the presence of a sound following a keypress. When a keypress triggered a 250 ms delayed sound (AS condition), a stronger keypress was associated with an earlier reported keypress time. When no sound followed a keypress (AO condition), a stronger keypress was associated with a later reported keypress time. The interpretation is that participants rely on the somatosensory feedback in the AO condition when reporting the keypress time. In the AS condition, both somatosensory feedback and the auditory feedback provide information about the keypress time. In this case, information from both sensory modalities is combined in the process of judging the keypress time. A strong keypress gains more weight for the somatosensory information than a light keypress, which leads to a reported keypress time that is closer to the somatosensory cue, i.e., a relatively early keypress time. The interpretation is further supported by a follow-up study showing that the action binding effect, i.e., the difference in the reported keypress time between the AS condition and the AO condition, was much stronger for light keypresses. This is because a light keypress leads to relatively early reported keypress time in the AO condition and late-reported keypress time in the AS condition, as compared to a strong keypress. In fact, the action binding effect was not even statistically significant with a one-tailed *t* test under strong keypress. In a similar vein, Wolpe and colleagues showed that increasing the intensity of the action triggered sound led to increased action binding (Wolpe et al. 2013).

Our finding has important implications for the understanding of the action binding effect. Action binding is defined as a forward shift of ‘the perceived time of intentional actions’ (Haggard et al. 2002). Actions, e.g., keypresses, however, do not occupy a time point, but are spread over a time period. The average duration between the computer-registered keypress onset time point and the computer-registered keypress offset time point in our dataset is 149 ms (study 1). The time from action-related EMG signal onset to the termination of action should be even longer (Haggard and Eimer 1999). Therefore, when asked to report the time of their action, participants may rely on some cues from the sensory feedback to infer the psychological time of the action [cf. the perceptual centre of a sound, Morton et al. (1976)]. In the AO condition, only the somatosensory feedback can provide information about the keypress time as the reported keypress time falls within the period when the somatosensory feedback is available. Other potential sources of information (e.g., motor intention) would lead to a much earlier reported keypress time. Furthermore, a significant positive correlation between the keypress peak force and the reported keypress time at the group level clearly suggests that the reported keypress time is constructed with information from the somatosensory feedback. It should be noted that the positive correlation result should not be interpreted in the sense that participants directly use the peak force to judge the keypress time in the AO condition. The peak force of a keypress is used to represent the somatosensory feedback of the keypress in the current study. Force parameters of a keypress (e.g., peak force, peak force latency, and keypress duration) are highly correlated (Ulrich and Wing 1991). At the moment, we are not sure which specific cue in the somatosensory feedback (or force parameter) is used to judge the keypress time, nor is it clear if the same cue is always used during the whole testing period. A clear understanding of how the somatosensory feedback influences the constructed keypress time is further complicated by the sensory processing delay. Nevertheless, from our results it is clear that somatosensory feedback is used in reporting the keypress time. Other factors, e.g., eye movements (since the keypress time was reported within the visual domain), might additionally influence the reported keypress time, which may be the reason why the average correlation coefficients in both AO and AS conditions were quite small (0.06 and − 0.06, respectively).

Based on the previous arguments, ‘the perceived time of intentional actions’ may rather be constructed from the timing of somatosensory cues in the AO condition, i.e., there is no action time for participants to perceive. This immediately suggests at least two other possible interpretations of action binding: (1) action binding may be due to a change in the parameter of the somatosensory cue between the AO and the AS conditions; (2) action binding may result from using different cues to infer the action time between the two conditions.

The first possibility may find support from the observation that distal sensory feedback (e.g., a sound) can reduce keypress intensity (Chase et al. 1961; Neszmelyi and Horvath 2018). Also in study 1 (no keypress force manipulation), keypresses in the AS condition (mean = 1.44 Newton; SD = 0.55) had lighter peak force than in the AO condition [mean = 1.72 Newton; SD = 0.94; *t*(41) = − 3.32, *p* = 9.38e−4, one-tailed, *d*_*z*_ = − 0.51]. The peak force latency in the AS condition (mean = 53.81 ms; SD = 15.80) was also slightly smaller than in the AO condition [mean = 57.20 ms; SD = 20.28; *t*(40) = − 2.39, *p* = 0.01, one-tailed, *d*_*z*_ = − 0.37]. However, a change of keypress force parameters cannot explain action binding. Theoretically, the decreased peak force in the AS condition should lead to an early reported keypress time, if the influence from the auditory feedback is not considered. However, AS condition has a late-reported keypress time in the action binding effect. Practically, matching the keypress peak force between conditions through trial selection (in study 1; Supplementary Figure 5) or experimental manipulation (in study 2) does not abolish the action binding effect. Although the force parameter change cannot explain the action binding effect, it has the potential to modulate the measured size of action binding. For example, if the physical action time is defined as the time point of peak force, the group average action binding effect will increase by ~ 3 ms (i.e., 57.20–53.81) in comparison to the calculation based on the computer-registered keypress time. This 3 ms increase, as shown already, is statistically significant.

The second possibility, i.e., different sensory cues are used to infer the action time, is supported by our data. If the somatosensory cue is used similarly in the AO and AS conditions, we should see a similar correlation pattern between the reported keypress time and the keypress peak force between the two conditions, which would imply that action binding is a change of perception towards the same event (the somatosensory cue). However, the reversed pattern of the correlation results indicates that the aforementioned scenario is unlikely. Rather, it suggests that somatosensory cue is still used in the AS condition, but in a different way. The best way to interpret the negative correlation between the reported keypress time and the keypress peak force, as we suggest, is to consider a competition between the somatosensory information and the auditory information in shaping the reported keypress time. Therefore, action binding should be understood as a perception change due to the change of the events being perceived. In this respect, we found that the excluded participants in the first study can provide additional support for this claim. As detailed in the methods section, some participants were excluded as they may have used the offset of the red circle as a cue to judge the keypress time. For these participants, no action binding can be found (Supplementary Figure 2; participants whose reported keypress time was less than − 100, i.e., more than 100 ms before the keypress time). This is most likely because they always used the same visual cue to judge the keypress time.

Most strikingly, the individual difference in action binding can be explained by the extent of competition between the somatosensory information and the auditory information, as indexed by the correlation between the keypress peak force and the reported keypress time in the AS conditions (negative correlations indicate strong competitions, Fig. 2e). A recent study by Lush and colleagues suggests that the reported action time can be modelled by a Bayesian process which combines cues that are informative of the action time (e.g., motor intention and action-induced sound) in a precision weighted manner (Lush et al. 2019). They showed that a high precision (the inverse of the squared standard deviation of within-participant timing judgement) in action time judgment was associated with a low action binding effect. This is largely consistent with our results. In the current study and the study by Wolpe et al. (2013), the strength of sensory information was experimentally manipulated. It is implicitly assumed that stronger sensory input from a specific modality (e.g., a strong keypress) would increase the signal-to-noise ratio of the information carried by the sensory input, and thereby leading to a decrease in the variance of reported action time, i.e., an increase in precision. Indeed, the average within-participant variability (standard deviation) of reported keypress time in strong keypress condition was found to be significantly lower than in light keypress condition. Therefore, we suggest that a high keypress peak force leads to a high signal-to-noise ratio of sensory information, which is manifested in the high precision of keypress time judgement and leads to reduced action binding effect. The multisensory information integration account of action binding, which is consistent with some recent progress in intentional binding research (Kirsch et al. 2019; Suzuki et al. 2019; Wolpe et al. 2013), contrasts with the endeavour to explain the individual difference in intentional binding (action binding and/or outcome binding) through high-level cognitive constructs such as the sense of agency (Moore and Obhi 2012). A sensory origin of action binding does not rule out the high-level cognitive explanation. However, it would imply a direct relationship between, e.g., sense of agency and multisensory information integration.

To conclude, the current work supports the idea that action binding results from a multisensory information integration process.

## Electronic supplementary material

Below is the link to the electronic supplementary material.

Supplementary material 1 (DOCX 782 kb)

## Data Availability

The data and materials for all experiments are available (10.6084/m9.figshare.8081495).

## Footnotes

1 A reviewer suggested repeating the correlation analysis by only including participants showing a positive action binding effect (i.e. *t* > 0). After excluding participants with negative t values, the correlation was not significant (Pearson’s *r* = − 0.02, *p* = 0.91, two-tailed, 95% CI = [− 0.45 0.43]). The reason for the change of correlation results is two folded: 1, a reduction of sample size from 42 to 32; 2, more importantly, only including positive *t* values led to a biased sampling of noise in the original dataset (i.e., noise leading to negative *t* values were inhibited), thereby the outliers in the original analysis cannot be detected anymore.
