# Supplementary material for: Action force modulates action binding: evidence for a multisensory information integration explanation
Source: Exp Brain Res. 2020 Jul 2;238(9):2019–29. doi: 10.1007/s00221-020-05861-4 (PMC7438375; doi:10.1007/s00221-020-05861-4)
Supplement: Supplementary file 1 — Supplementary material 1 (DOCX 782 kb) [file 221_2020_5861_MOESM1_ESM.docx]

*Supplementary materials for*

**Action force modulates action binding: Evidence for a multisensory information integration explanation**

Liyu Cao, Michael Steinborn, Wilfried Kunde, & Barbara Haendel

***Corresponding author:**

Dr. Liyu Cao

Department of Psychology (III)

Julius-Maximilians-Universität Würzburg

97070, Würzburg, Germany

Tel:  +49 931 318 6838

E-mail: liyu.cao@uni-wuerzburg.de

Submitted to: Experimental Brain Research


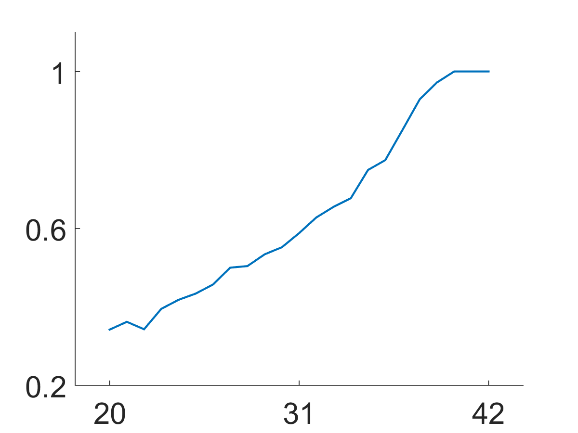


Number of participants

Statistical power

**Supplementary Figure 1** Statistical power of study 2 estimated with data acquired from study 1. The bootstrap analysis showed that the statistical power (probability of detecting the predicted interaction effect) increases with the number of participants included in the study. When the participant number reaches 40, the statistical power is 1.

Mean reported time (AO), ms

Mean reported time (AS), ms


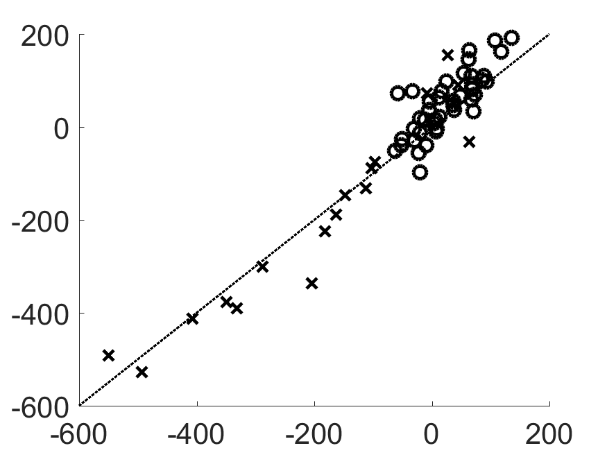


**Supplementary Figure 2** The full dataset from study 1. Excluded participants are marked with crosses and included participants are marked with circles. Note that the action binding effect is also significant with all participants included (*t*(59) = 2.54, *p* = 0.007, one-tailed, *d_z_* = 0.33). The straight line is the diagonal line.


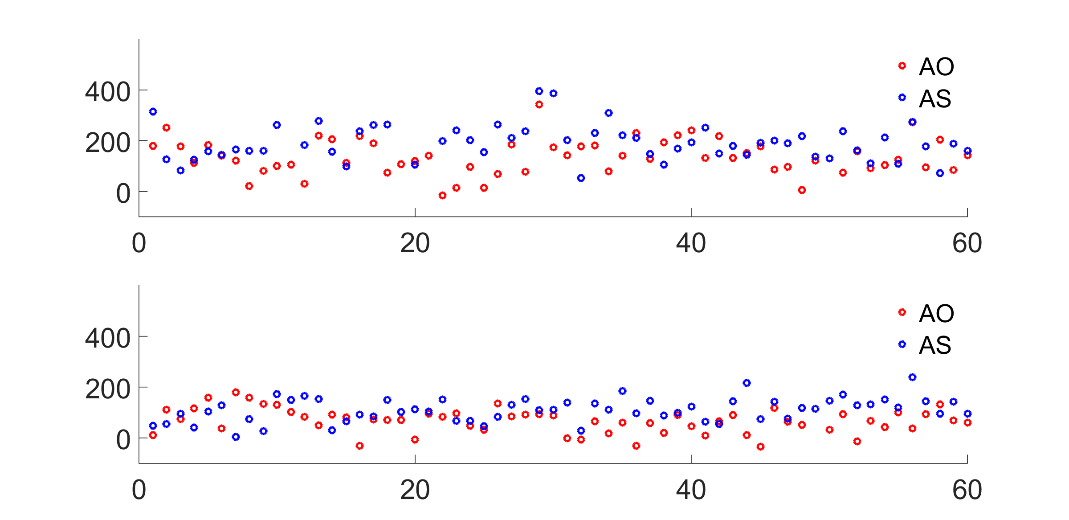


Trial number

Reported keypress time, ms

Subject 1

Subject 2

**Supplementary Figure 3** The t value represents the size of action binding better than the raw difference in reported keypress time. Each dot in the figure represents the reported keypress time in a single trial. Taking the raw difference in reported keypress time between the AS and AO conditions, the action binding effect was 54.99 ms for subject 1 and 43.04 ms for subject 2. Taking the t-value, the action binding effect was 4.25 for subject 1 and 4.93 for subject 2. In this case, the t-value and raw difference lead to different conclusion when comparing the size of action binding effect between the two example participants (however, both measures indicate that the two participants had very strong action binding effects). Which one makes more sense? In our opinion, the result indicated by the t-value may be closer to the truth. As the figure shows here, subject 2 had a very small variance in the reported keypress time over trials, i.e. a very high precision. Therefore, the reported keypress time from subject 2 should be given a high weight (in analogy to the Bayesian principle), which is implemented through the variance normalisation, i.e. calculating the t-value.


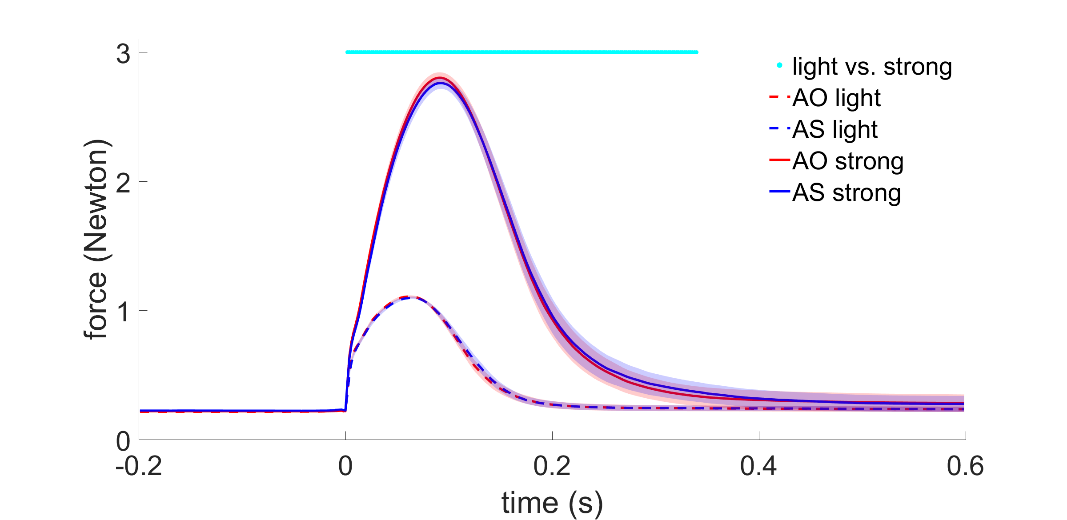


**Supplementary Figure 4** The average keypress force trajectory in study 2. For each condition of each participant, the keypress force trajectories of individual trials were aligned to the time point when the keypress force started to increase (time 0 in the figure), and the averaged. The figure shows the group average of keypress force trajectory (shading indicates ± 1 standard error). The force was compared between the light keypress condition (average of AO light and AS light) and the strong keypress condition (average of AO strong and AS strong) at each time point from -200 ms to 600 ms using a within-subjects t-test. The time points showing a significant difference were marked by the cyan line ([1 340] ms; multiple comparisons were corrected using the cluster correction; Maris & Oostenveld (2007), Journal of Neuroscience Methods, 164(1), 177-190).


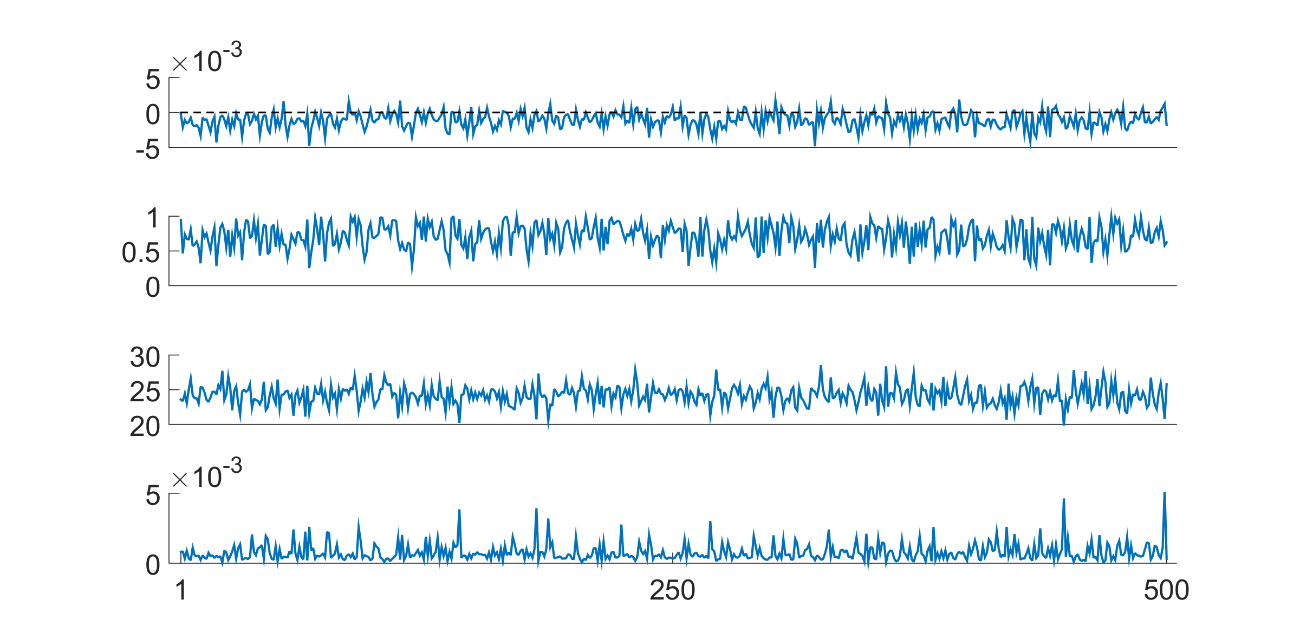


Repetition

*p* value

Force difference (AS - AO)

*p* value of Force comparison

Action binding effect (AS - AO)

*p* value of action binding effect

*N*

*ms*

*p* value

**Supplementary Figure 5** Matching the keypress peak force in AO and AS conditions in study 1. The keypress peak force matching between AO and AS conditions was performed as the following: The full force range (in raw unit) was divided into small windows with a length of 25 (e.g. 51 to 75, 76 to 100,…). For each force window, the trials with peak force lying within the window from both conditions were selected. If one condition had more trials than the other in a force window, the trial number was matched between conditions through random selection. The above procedure was repeated 500 times for each of the 42 participants in study 1. For each of the 500 repetitions, there were an average of 31.67 (SD = 8.66) trials selected from each condition. The first row shows the group average peak force difference between AS and AO from the 500 repetitions. The force difference is very small and no significant difference can be found from a paired t-test (second row shows the *p* value of the t-test). The third row shows the group average action binding effect and the corresponding *p* value from a paired-test is shown in the fourth row. Therefore, when the keypress peak force is matched between conditions in study 1, action binding is still present.
